# Emotion Regulation in Everyday Life: The Role of Goals and Situational Factors

**DOI:** 10.3389/fpsyg.2020.00877

**Published:** 2020-05-19

**Authors:** Rafael Wilms, Ralf Lanwehr, Andreas Kastenmüller

**Affiliations:** ^1^Department of Education Studies and Psychology, University of Siegen, Siegen, Germany; ^2^Department of International Management, South Westphalia University of Applied Sciences, Meschede, Germany

**Keywords:** situational factors, emotion regulation, emotion regulation goals, experience sampling study, negative emotions

## Abstract

This study addresses three questions: How often and how consistently do predictors for emotion regulation choice occur in daily life? What predicts emotion regulation choice in daily life? How do predictors for emotion regulation choice interact in daily life? We examined emotion regulation goals (i.e., prohedonic and social goals), situational factors (i.e., perceived control, expected reoccurrence, and emotional intensity), and emotion regulation strategies (i.e., active coping, distraction, rumination, cognitive reappraisal, and expressive suppression) in negative emotion events. A total of 110 individuals (65% female) participated in an experience sampling study and received beeps, five times a day over the course of 9 days. We used a random intercept model to estimate our results. Emotion regulation goals and situational factors vary strongly in different events within the same person. Emotion regulation strategies, effective in changing the emotional experience, are crucial for prohedonic goals, whereas expressive suppression is important for social goals. Perceived control was positively associated with putatively adaptive strategies. Emotional intensity and expected reoccurrence were negatively associated with putatively adaptive strategies. Emotional intensity was positively associated with putatively maladaptive strategies. Emotion regulation strategies were not associated with the interaction of emotion regulation goals and situational factors. We conclude that emotion regulation goals and situational factors are extremely context-dependent, suggesting that they should be treated as states. Emotion regulation goals appear to have a functional association with strategies for prohedonic and social goals. The associations between situational factors and strategies in daily life appear to be largely different from the results found in the laboratory, emphasizing the importance of experience sampling studies.

## Introduction

The way we regulate our emotions has important implications for our well-being (Webb et al., 2012) and our social relationships (Cameron and Overall, 2017). Adaptive emotion regulation requires a flexible alternation between strategies in accordance with personal goals and contextual demands (Bonanno and Burton, 2013; Aldao et al., 2015). Emotion regulation goals (e.g., the goal of feeling better) and situational factors (e.g., emotional intensity) influence the choice for or against particular emotion regulation strategies (Sheppes et al., 2011; Haines et al., 2016; Millgram et al., 2019). These phenomena were primarily investigated in the lab (e.g., Millgram et al., 2019). Even though first studies examined them in daily life (e.g., Haines et al., 2016; English et al., 2017), we are far from having a complete picture of emotion regulation in daily life. We want to contribute to this picture by shedding light on three questions: How often and how consistently do predictors for emotion regulation choice occur in daily life? What predicts emotion regulation choice in daily life? How do predictors for emotion regulation choice interact in daily life?

To address these questions, we conducted an experience sampling study (Beal, 2015) and examined emotion regulation goals, situational factors, and emotion regulation strategies five times a day at negative emotion events. In detail, we included a prohedonic goal, four social goals (i.e., *to avoid conflict*, *to keep up appearances*, *to make others feel better*, and *to influence others*), and three situational factors (perceived control over the situation, emotional intensity, and expected reoccurrence of a similar situation) as predictors. As criterions, we used emotion regulation strategies from the entire emotion generative process (Gross, 1998): situation selection/modification (i.e., active coping), attentional deployment (i.e., distraction and rumination), cognitive change (i.e., cognitive reappraisal), and response-modulation (i.e., expressive suppression).

To the authors’ knowledge, this is the first study that examines emotion regulation goals and situational factors in combination at negative emotion events. We focus on negative events, as individuals regulate negative emotions more often than positive ones (Brans et al., 2013; English et al., 2017), predictors of emotion regulation have only rarely been studied in daily life in general, and the regulation of negative emotions offers a more solid foundation for theoretical reasoning. Moreover, the current study has a higher resolution of daily life and also collects events with lower emotional intensity. Prior studies (e.g., English et al., 2017; Kalokerinos et al., 2017a) examined the most negative event of the day, whereas we focus on the most negative event of the last 3 h. Thus, this study captures a more realistic picture of emotion regulation in daily life.

### Emotion Regulation Goals

Individuals often regulate their emotions to pursue their goals (Tamir et al., 2015; Millgram et al., 2019). Goals are a cognitive representation of an end state (Fishbach and Ferguson, 2007). They typically come up when an individual becomes aware of the discrepancy between a current state and a desired end state (Carver and Schreier, 2000). The detected discrepancy usually motivates people to reduce this gap. The current study investigates two different classes of emotion regulation goals: prohedonic goals and social instrumental goals (Tamir, 2016). These are important to examine because they often occur in everyday life (English et al., 2017; Eldesouky and English, 2018). Prohedonic goals aim at changing the *current* pleasure-to-pain ratio in favor of pleasure, and thus aim to change the feeling component (e.g., experienced pleasure or pain) of an emotion. By contrast, instrumental goals encompass superordinate goals that are potentially, but not necessarily achieved by regulating emotions (Tamir, 2016). One particular class of instrumental goals are social goals, which reflect the pursuit of creating and maintaining positive social relationships (Gable and Berkman, 2008; Netzer et al., 2015; Tamir, 2016). For instance, it may be beneficial to signal empathy for a friend whose girlfriend has left him.

### Situational Factors

Situations, in which individuals regulate their emotions, may differ dramatically from each other (Bonanno and Burton, 2013; Aldao et al., 2015; Doré et al., 2016). For example, individuals may regulate their emotions in situations of weak or strong emotional intensity. We refer to specific situational differences as situational factors. Previous studies showed that situational factors affect emotion regulation choice. For example, most individuals prefer cognitive reappraisal (distraction) over distraction (cognitive reappraisal) in situations of low (high) emotional intensity (Sheppes et al., 2011). Since situational factors often determine how individuals regulate their emotions, they are crucial for understanding emotion regulation in daily life.

### How Often and How Consistently Do Predictors for Emotion Regulation Choice Occur in Daily Life?

In order to understand emotion regulation in daily life, it is important to understand not only what predicts emotion regulation choices, but also how often and consistently these predictors occur. A strong predictor that occurs very seldom may be less important than a weaker predictor that occurs frequently. Likewise, a predictor that occurs consistently for some people, but not for others may reveal group differences in emotion regulation choice.

Prior daily diary studies examined the fluctuation of emotion regulation goals. They demonstrated that prohedonic goals are the primary reason for emotion regulation, but that social goals also play an important role (English et al., 2017; Kalokerinos et al., 2017c; Eldesouky and English, 2018). These studies examined emotion regulation goals only once a day and targeted the strongest negative event of that day (e.g., English et al., 2017; Kalokerinos et al., 2017b). Consequently, we only have a preliminary understanding of how often and consistently emotion regulation goals occur.

### What Predicts Emotion Regulation Choice in Daily Life?

The emotion regulation flexibility framework (Bonanno and Burton, 2013; Aldao et al., 2015) challenges that certain strategies are uniformly more adaptive or efficacious than others and argues instead that a strategy’s effectiveness depends on the context. Consistently, individuals show considerable variability in strategy use in everyday life (Blanke et al., 2019). In the following, we develop hypotheses about contexts (i.e., emotion regulation goals and situational factors) that affect the choice for or against a particular emotion regulation strategy.

#### Hedonic Goals and Emotion Regulation

Individuals expect strategies, effective in changing the emotional experience (Webb et al., 2012), to be more potent for achieving prohedonic goals (Sheppes et al., 2014; Millgram et al., 2019). For example, distraction, instead of rumination, is primarily used when individuals are asked to reduce their negative emotional experience (Millgram et al., 2019).

Based on that, we propose that prohedonic goals are positively associated with active coping, distraction, and cognitive reappraisal, as those are effective in changing the current emotional experience (Webb et al., 2012, 2018). By contrast, prohedonic goals are negatively associated with rumination, since it is effective in increasing or maintaining the negative emotional experience (Webb et al., 2012; Millgram et al., 2019). However, people often believe that this strategy will help them to feel better (Nolen-Hoeksema et al., 2008). So, mixed evidence exists for the relationship between prohedonic goals and rumination. Finally, expressive suppression should not be associated with prohedonic goals (English et al., 2017) because it is not effective in changing the emotional experience (Webb et al., 2012).

H1: Prohedonic goals are positively associated with active coping.
H2: Prohedonic goals are positively associated with distraction.
H3: Prohedonic goals are positively associated with cognitive reappraisal.

#### Social Goals and Emotion Regulation

We focus on four social goals: *to avoid conflict*, *to keep up appearances*, *to make others feel better*, and *to influence others*.

*To keep up appearances* requires hiding an emotion from at least one other person. Accordingly, we assume that the goal is positively associated with expressive suppression, as it decreases the degree of facial expression of an emotion (Webb et al., 2012), and positively associated with distraction, as it decreases the emotional experience (Webb et al., 2012), which in turn (potentially) decreases its expression (Gross et al., 2000).

H4: *To keep up appearances* is positively associated with expressive suppression.
H5: *To keep up appearances* is positively associated with distraction.

*To avoid conflict with others* requires a down-regulation of an emotion experience or its expression. For example, experiencing or expressing anger may increase the likelihood of conflict (e.g., Kassinove et al., 2002). As distraction and expressive suppression effectively decrease the experience and the expression of emotions, respectively (Webb et al., 2012), we assume that *to avoid conflict with others* is positively associated with these strategies.

H6: *To avoid conflict with others* is positively associated with expression suppression.
H7: *To avoid conflict with others* is positively associated with distraction.

*To make others feel better* primarily targets emotions of others, and we included only expressive suppression as an interpersonal emotion regulation strategy. However, expressive suppression was not associated with *to make others feel better* in another study (English et al., 2017); we therefore do not expect the goal *to make others feel better* to predict any included strategy.

*To influence others* requires the passing of information to others, as the transmitter can only influence others if information is passed to the receiver. Expressive suppression reduces the emotional information passed (Greenaway and Kalokerinos, 2017; Kalokerinos et al., 2017a), as it diminishes the emotional expression of the transmitter (Webb et al., 2012). Accordingly, we assume that *to influence others* is negatively associated with expressive suppression.

H8: *To influence others* is negatively associated with expressive suppression.

#### Situational Factors and Emotion Regulation

We focus on three characteristics of situations: Individuals may perceive different levels of control over the situation (referred to as perceived control; Haines et al., 2016), experience different degrees of emotional intensities (referred to as emotional intensity; Dixon-Gordon et al., 2015), or perceive different likelihoods of the event repeating itself (referred to as expected reoccurrence; Sheppes et al., 2014). Those situational factors influence emotion regulation choice.

Perceived control may increase the likelihood of using active coping, as trying to change a situation requires at least some control over the situation. By contrast, it may decrease the likelihood of using cognitive reappraisal (Troy et al., 2013; Haines et al., 2016), as reappraising may be more useful, when a situation cannot be changed. For example, if a woman is in a toxic relationship, it may be more detrimental to reappraise the behavior of her boyfriend, compared to actively taking actions (e.g., leaving him). Accordingly, we assume that perceived control is positively associated with active coping and negatively associated with cognitive reappraisal.

H9: Perceived control is positively associated with active coping.
H10: Perceived control is negatively associated with cognitive reappraisal.

Emotional intensity influences whether individuals choose to engage in distraction or cognitive reappraisal (Sheppes et al., 2014). In situations with low (high) emotional intensity, most individuals prefer to choose cognitive reappraisal (distraction) over distraction (cognitive reappraisal) (Sheppes et al., 2014; Milyavsky et al., 2019). Further, emotional intensity is positively associated with the use of rumination and active coping (Dixon-Gordon et al., 2015; Van Bockstaele et al., 2019). Accordingly, we expect emotional intensity to be positively associated with active coping, distraction, and rumination, but negatively associated with cognitive reappraisal.

H11: Emotional intensity is positively associated with active coping.
H12: Emotional intensity is positively associated with distraction.
H13: Emotional intensity is positively associated with rumination.
H14: Emotional intensity is negatively associated with cognitive reappraisal.

Expected reoccurrence influences whether individuals choose distraction or cognitive reappraisal. In situations with low (high) expected reoccurrence, individuals choose distraction (cognitive reappraisal) over cognitive reappraisal (distraction). Cognitive reappraisal changes the emotional experience of a stimulus in the long-term, whereas distraction only changes it in the short-term (Sheppes et al., 2014). Similarly, active coping changes (potentially) the long-term emotional reaction to a stimulus by altering it (Gross, 1998; Van Bockstaele et al., 2019). If a person expects to be confronted with the same stimuli again, cognitive reappraisal and active coping are more beneficial than distraction, as they reduce the negative impact of the stimulus. Accordingly, we assume expected reoccurrence to be positively associated with active coping and cognitive reappraisal, but negatively associated with distraction.

H15: Expected reoccurrence is positively associated with active coping.
H16: Expected reoccurrence is positively associated with cognitive reappraisal.
H17: Expected reoccurrence is negatively associated with distraction.

### How Do These Predictors Interact in Daily Life?

We propose that the interaction of emotion regulation goals and situational factors predicts the preference for emotion regulation strategies. Our predictions focus on prohedonic goals, as they provide a more solid foundation for our theoretical reasoning (e.g., Sheppes et al., 2014; Millgram et al., 2019; Tamir et al., 2019).

The effect of prohedonic goals on distraction and cognitive reappraisal (Sheppes et al., 2014) is moderated by emotional intensity. That is, we expect that the effect of prohedonic goals on distraction and cognitive reappraisal to be stronger (weaker) with a high (low) emotional intensity.

H18: The positive relationship between prohedonic goals and distraction is moderated by emotional intensity, so that the effect is stronger with a higher emotional intensity and weaker with a lower emotional intensity.
H19: The positive relationship between prohedonic goals and cognitive reappraisal is moderated by emotional intensity, so that the effect is stronger with a higher emotional intensity and weaker with a lower emotional intensity.

The effect of prohedonic goals on active coping, cognitive reappraisal, and distraction is moderated by expected reoccurrence. As cognitive reappraisal and active coping change the reaction to a negative stimulus in the long term, we assume that individuals prefer them, when they want to feel better and the expected reoccurrence is high instead of low. As distraction changes the reaction to a negative stimulus in the short term, we assume that individuals avoid it, when they want to feel better and the expected reoccurrence is low instead of high.

H20: The positive relationship between prohedonic goals and active coping is moderated by expected reoccurrence, so that the effect is stronger with a higher expected reoccurrence and weaker with a lower expected reoccurrence.
H21: The positive relationship between prohedonic goals and distraction is moderated by expected reoccurrence, so that the effect is weaker with a higher expected reoccurrence and stronger with a lower expected reoccurrence.
H22: The positive relationship between prohedonic goals and cognitive reappraisal is moderated by expected reoccurrence, so that the effect is stronger with a higher expected reoccurrence and weaker with a lower expected reoccurrence.

## Materials and Methods

### Participants and Procedure

The participants were 143 students in business-related study programs from three universities in Germany (65% female and 35% male; age *M* = 21.81 and *SD* = 2.52) and were recruited by advertising the study at the start of different courses.

The experience sampling study had two parts: The introduction session and the experience sampling phase. First, participants signed the informed consent form and completed different questionnaires unrelated to this study. Second, the authors explained the procedure of the experience sampling study to the participants and outlined how to install the app, RealLifeExp Version 2.5.2, on the participants’ own smartphones. Third, the participants were trained on the experience sampling study’s protocol.

The participants received beeps at 10 a.m., 1, 4, 7, and 10 p.m. over a period of 9 consecutive days. The participants had to answer the beep within 1.5 h; otherwise, the beep was counted as not-responded. Aiming to reduce the burden of participants (Bolger et al., 2003), we chose these time periods based on information by students from the same universities. Those did not participate in this study. The median response time was 06:22 s (*M* = 14:58 s; *SD* = 19:58 s). To incentivize participants, they received a €10 Amazon voucher if they responded to at least 26 beeps.

We excluded participants who did not respond to at least 20 beeps to ensure that participants had sufficient motivation for the experience sampling study (Podsakoff et al., 2012). On average, participants completed 37.92 beeps. One hundred ten participants remained.

### Measures

At each beep, items were asked in the following order. First, participants were thanked for their participation, asked about their mood, what they have done since the last beep (or at the first beep, since they have been awake), and if they experienced a negative emotion within the last 3 h. Only if they responded with *yes* to the last question were they instructed to report on the event that elicited the most intense negative emotions, asked which emotion they experienced (unrelated to this study), and answered questions about situational factors, emotion regulation strategies, emotion regulation goals, and emotion regulation direction (unrelated to this study). In 718 of 4171 situations (17.21%), participants reported they experienced at least one negative emotion during the last 3 h.

#### Situational Factors

Participants were asked to indicate three situational factors. We measured the emotional intensity of the focal event (“How intense was your most negative emotion?”), their perceived control (“To what extent was the event in which you felt the negative emotion(s) under your control?”), and the expected reoccurrence (“How likely is it that this situation will repeat itself or that a very similar one will occur?”). Each of these questions appeared on a separate page. All situational factors were measured on a scale from not at all (1) to very (7).

#### Emotion Regulation Strategies

Participants were asked to recall the situation that elicited the negative emotion(s) and indicate how intensively they used the following five emotion regulation strategies (e.g., Kalokerinos et al., 2017b): active coping (“I have taken active steps to improve the situation”; Knoll et al., 2005), distraction (“I distracted myself from the situation or my feelings”), rumination (“I dwelled on the situation or my feelings”), cognitive reappraisal (“I tried to change my perspective on the situation or to change how I think about it”), and expressive suppression (“I suppressed my outward expression of my emotions”). All emotion regulation strategies were presented in one block and measured on a scale from not at all (1) to very (7).

#### Emotion Regulation Goals

Participants were asked to recall the situation that elicited the negative emotion(s) again and indicate the emotion regulation goals, they pursued in this situation. The questions were introduced with “*I tried to influence my emotions …*,” and were largely oriented on the study by English et al. (2017): a prohedonic goal (“to make me feel better”), four social goals (“to avoid conflict,” “to keep up appearances,” “to make others feel better,” and “to influence others”) and one performance goal (“to get work done or to learn”; used as a control variable). Further, participants could choose to indicate “other” and “I did not want to influence my emotions.” All emotion regulation goals were presented in one block and were dichotomous (0 = not chosen; 1 = chosen) to reduce the participants’ burden (Bolger et al., 2003). Participants were free to choose how many goals they endorsed.

## Data Analysis and Results

We structure the data analysis and the results in three questions: How often and how consistently do predictors for emotion regulation choice occur in daily life? What predicts emotion regulation choice in daily life? How do predictors for emotion regulation choice interact in daily life? All analysis were performed with R Cran (R Core Team, 2015). The data is available in the Supplementary Material.

### How Often and How Consistently Do Predictors for Emotion Regulation Choice Occur in Daily Life?

#### Data Analysis

We calculated percentages based on regular averages and on averages of the person-means. The average of the person-means^1^ weighs the response of each person equally and is more robust against different numbers of events per person.

We calculated the ICCs based on a three-level model (events within days within persons) and as the ratio of between-variance to total variance (i.e., the sum of between-variance, day-variance, and event-variance; Baguley, 2012). They can be interpreted as follows: A low ICC indicates that the variance observed in a variable is determined by the event and the day; the pattern of the variable varies a lot within individuals. A high ICC indicates that the variance is determined by the individual; in other words, the pattern of the variable is very similar among different events and days within one individual, but very different among individuals.

#### Results

Table 1 shows the number of events with the goals endorsed, the percentages of events with this goal, based on regular averages and based on averages of the person-means, and the ICCs for emotion regulation goals. The goals *to feel better*, *to keep up appearances*, and *to avoid conflict* were most frequent, whereas *to make others feel better* and *to influence others* were the least frequent. The patterns of the regular averages and the averages of the person-means seem to be very similar, showing consistency between individuals with higher vs. lower response rates.

**TABLE 1:** Descriptive statistics on emotion regulation goals.

**Goals**	***n* events with this goal**	**% events with this goal**	**% events with this goal based on person-means**	**ICC**
To feel better	266	0.37	0.34	0.21
To avoid conflict with others	175	0.24	0.24	0.11
To keep up appearances	213	0.30	0.24	0.14
To make others feel better	65	0.09	0.08	0.09
To influence others	17	0.02	0.02	0.02

**The abbreviations n and ICC refer to the sample size and intraclass coefficient, respectively.**

The ICCs show that emotion regulation goals vary strongly from situation to situation within one person. The most stable goal is *to feel better* with 21% between-person variance. The goals *to keep up appearances* and *to avoid conflict* have a similar level of between-person variance. The goals *to make others feel better* and *to influence others* vary more dramatically from situation to situation.

Table 2 shows the means, standard deviations, and ICCs for situational factors. In descending order, expected reoccurrence, emotional intensity, and perceived control show the highest level of intensity. The patterns of the regular averages and averages of the person-means seem to be very similar, showing consistency between individuals with higher vs. lower response rates.

**TABLE 2:** Descriptive statistics on situational factors.

**Situational factors**	***M***	***SD***	***M* of person-means**	***SD* of group mean**	**ICC**
Perceived control	3.54	2.00	3.55	1.42	0.28
Emotional intensity	4.48	1.54	4.42	1.01	0.17
Expected reoccurrence	5.25	1.65	5.04	1.16	0.22

**The abbreviations M, SD, and ICC refer to mean, standard deviation, and intraclass coefficient, respectively.**

The ICCs show that situation factors vary substantially more among the events and days than among individuals. Perceived control varies the least and emotional intensity the most among events and days.

### What Predicts Emotion Regulation Choice in Daily Life?

#### Data Analysis

Accounting for the nested structure of our experience sampling data, we conducted multilevel analyses using *lme4* (Bates et al., 2015). We are primarily interested in the within-person effects, and therefore included the person-means (between-person effects) of each independent variable. Those person-means absorb all interindividual differences that may affect within-person effects (Mundlak, 1978).

The within-person effect can be interpreted as a situation specific effect. For example, if a person wants to feel better, the goal may affect choosing cognitive reappraisal in the focal situation but does not provide information about the relationship between habitual prohedonic goals and the habitual use of cognitive reappraisal.

To estimate our results, we included the emotion regulation goals *to feel better*, *to avoid conflict*, *to keep up appearances*, *to make others feel better*, *to influence others*, and *to get work done or learn* as well as the situational factors *expected reoccurrence*, *emotional intensity*, and *perceived control* simultaneously as predictors. We used active coping, distraction, rumination, cognitive reappraisal, and expressive suppression as criterions in separate models.

Following the recommendation by Luke (2017) we estimated our linear mixed effects model with the robust maximum likelihood estimator and estimated the degrees of freedom with the Kenward-Roger approximation (Kenward and Roger, 1997) using the package *pbkrtest* (Halekoh and Højsgaard, 2014). We modeled three-level random intercept models (events within days within persons), unless indicated otherwise and used standardized variables to obtain standardized effects.

In case a model suffers from heteroskedasticity, we fitted it again with the package *robustlmm* (Koller, 2016). The package allows a more robust estimation of standard errors than the normal *lme4* package. The function *rlmer* reduces the influence of outliers that may cause heteroskedasticity. Since there is no package available that provides degrees of freedom for this robust estimation, we followed the t-as-z approach. To make our heuristic approach less susceptible to alpha errors, we decided to increase the critical *p-*value to 0.01 (*t-*value of |2.236|) (for a similar approach, see Kornilov et al., 2019).

Finally, we computed the explained within-variance based on the $R_{1}^{2}\left(approx.\right)$, developed by Bryk and Raudenbush (1992), which showed a good estimation performance in previous simulation studies (LaHuis et al., 2014).

#### Results

Table 3 shows the results of what predicts emotion regulation strategies, whereas Table 4 provides an overview of the supported or unsupported hypotheses. Active coping was positively associated with *to feel better* [H1; β = 0.09; *F*(1, 615.37) = 5.01; *p* = 0.03] and perceived control [H9; β = 0.09; *F*(1, 615.52) = 4.20; *p* = 0.04]. Contrary to our hypotheses, active coping was negatively associated with emotional intensity [H11; β = −0.14; *F*(1, 611.61) = 12.69; *p* < 0.00] and expected reoccurrence [H15; β = −0.09; *F*(1, 611.59) = 4.81; *p* = 0.03]. The predictors jointly explained the within-variance of active coping [$R_{1}^{2}\left(approx.\right)$ = 0.07].

**TABLE 3:** Emotion regulation goals and situational factors as joint predictors for emotion regulation strategies in random intercept models.

	**Active coping**	**Distraction**	**Rumination**	**Cognitive reappraisal**	**Expressive suppression**
Intercept	–0.01	–0.03	–0.01	0.01	–0.06
**Emotion Regulation Goals**					
To feel better	0.09^∗^	0.19^∗∗∗^	0.10^∗∗^	0.12^∗∗∗^	0.04
To avoid conflict	0.05	0.12^∗∗^	0.03	0.07	0.21^∗∗∗^
To keep up appearances	–0.08	0.11^∗∗^	0.07	0.04	0.40^∗∗∗^
To make someone else feel better	0.03	0.10^∗∗^	0.03	0.09^∗^	0.03
To influence others	0.08^∗^	−0.07^∗^	0.01	0.02	–0.05
**Situational Factors**					
Perceived control	0.09^∗^	0.03	0.00	0.13^∗∗^	0.06
Emotional intensity	–0.14^∗∗∗^	–0.07	0.30^∗∗∗^	–0.11^∗∗^	−0.08^∗^
Expected reoccurrence	−0.09^∗^	–0.04	0.01	–0.12^∗∗^	–0.02
**Interaction Effects**					
To work or learn	0.03	0.02	–0.01	0.04	0.02
To feel better ^∗^ emotional intensity		0.00		–0.01	
To feel better ^∗^ expected reoccurrence	0.00	0.02		–0.05	

**^∗^p < 0.05. ^∗∗^p < 0.01. ^∗∗∗^p < 0.001. Effects reported as standardized regression estimates based on the random intercept model. Significance is estimated by using Kenward–Rogers approximation (Kenward and Roger, 1997), and given the presence of heteroskedasticity, it is based on robust estimation (Koller, 2016). The model included person-means of the focal variables, but for simplicity, we omitted them in the table.**

**TABLE 4:** Overview of the supported or unsupported hypotheses.

	**Active coping**	**Distraction**	**Rumination**	**Cognitive reappraisal**	**Expressive suppression**
To feel better	✓ (+/+)	✓ (+/+)	(?/+)	✓ (+/+)	–
To avoid conflict	–	✓ (+/+)	–	–	✓ (+/+)
To keep up appearances	–	✓ (+/+)	–	–	✓ (+/+)
To influence others	–	–	–	–	× (−/0)
Perceived control	✓ (+/+)	–	–	× (−/+)	–
Emotional intensity	× (+/−)	× (+/−)	✓ (+/−)	✓ (−/−)	–
Expected reoccurrence	× (+/−)	× (+/−)	–	× (+/−)	–
To feel better ^∗^ emotional intensity	–	×	–	×	–
To feel better ^∗^ expected reoccurrence	×	×	–	×	–

**The expected/observed direction of the main effects are provided in parentheses. Dashes indicate no hypothesis.**

Testing the hypotheses for distraction, the day component of the model yields zero variance. Accordingly, we fitted a two-level model (events within persons) for distraction. Distraction was positively associated with *to feel better* [H2; β = 0.19; *F*(1, 611.75) = 24.11; *p* < 0.00], *to keep up appearances* [H5; β = 0.11; *F*(1, 611.75) = 8.14; *p* < 0.00], and *to avoid conflict* [H7; β = 0.12; *F*(1, 611.75) = 10.28; *p* < 0.00]. Contrary to our hypotheses, distraction was not associated with emotional intensity [H12; β = −0.07; *F*(1, 611.75) = 3.58; *p* = 0.06] and expected reoccurrence [H17; β = −0.04; *F*(1, 611.75) = 1.23; *p* = 0.27]. Since this model suffered from heteroskedasticity, we also fitted a model with the package *robustlmm* (Koller, 2016). The results of the robust model remained essentially unchanged, except that the non-robust model showed an unexpected and significant positive relationship for *to influence others*, but the robust model did not. The predictors jointly explained the within-variance of distraction [$R_{1}^{2}\left(approx.\right)$ = 0.08].

Rumination was positively associated with emotional intensity [H13; β = 0.30; *F*(1, 582.35) = 80.42; *p* < 0.00] and *to feel better* [β = 0.10; *F*(1, 603.15) = 7.80; *p* < 0.01]. The predictors jointly explained the within-variance of distraction [$R_{1}^{2}\left(approx.\right)$ = 0.13].

Cognitive reappraisal was positively associated with *to feel better* [H3; β = 0.12; *F*(1, 610.47) = 9.08; *p* < 0.00] and emotional intensity [H14; β = −0.11; *F*(1, 609.06) = 7.45; *p* = 0.01]. Contrary to our hypotheses, cognitive reappraisal was positively associated with perceived control [H10; β = 0.13; *F*(1, 610.44) = 8.92; *p* < 0.00] and negatively associated with expected reoccurrence [H16; β = −0.12; *F*(1, 604.38) = 9.05; *p* < 0.00]. Since this model suffered from heteroskedasticity, we estimated a robust model (Koller, 2016). The results remained essentially unchanged. The predictors jointly explained the within-variance of cognitive reappraisal [$R_{1}^{2}\left(approx.\right)$ = 0.06].

Expressive suppression was positively associated with *to keep up appearances* [H4; β = 0.40; *F*(1, 617.81) = 128.08; *p* < 0.00] and *to avoid conflict* [H6; β = 0.21; *F*(1, 618.04) = 35.56; *p* < 0.00]. Contrary to our hypotheses, it was not negatively associated with *to influence others* [β = −0.05; *F*(1, 616.97) = 2.50; *p* = 0.11]. The predictors jointly explained the within-variance of expressive suppression [$R_{1}^{2}\left(approx.\right)$ = 0.22].

Testing the assumptions of linear mixed effects model, we checked the normality assumption of residuals visually, heteroskedasticity, and tested for multicollinearity (see Table A1 for the within-correlations of the predictors). Those assumptions did not seem to be violated.

### How Do Predictors for Emotion Regulation Choice Interact in Daily Life?

#### Results

Table 3 shows the results of the interaction of emotion regulation goals and situational factors. The interaction between emotional intensity and *to feel better* was not positively associated with distraction [H18; β = 0.00; *F*(1, 662.67) = 0.01; *p* = 0.93]. The interaction between emotional intensity and *to feel better* was not negatively associated with cognitive reappraisal [H19; β = −0.01; *F*(1, 653.13) = 0.09; *p* = 0.76].

The interaction between expected reoccurrence and *to feel better* was not positively associated with active coping [H20; β = 0.00; *F*(1, 673.33) = 0.00; *p* = 0.97]. The interaction between expected reoccurrence and *to feel better* was not positively associated with distraction [H21; β = 0.02; *F*(1, 659.84) = 0.44; *p* = 0.51]. The interaction between expected reoccurrence and *to feel better* was not positively associated with cognitive reappraisal [H22; β = −0.05; *F*(1, 667.72) = 3.37; *p* = 0.07].

## Discussion

As the choice of emotion regulation strategies has a profound impact on our well-being (Webb et al., 2012) and our social lives (Cameron and Overall, 2017), it is crucial to understand its antecedents. This study addressed three questions: How often and how consistently do predictors for emotion regulation choice occur in daily life? What predicts emotion regulation choice in daily life? How do predictors for emotion regulation choice interact in daily life? Moreover, it complements earlier research, as it examines emotion regulation goals and situational factors in combination and captures a broader range of emotional intensities, compared to other studies (English et al., 2017; Kalokerinos et al., 2017c).

### How Often and How Consistently Do Predictors for Emotion Regulation Choice Occur in Daily Life?

#### Emotion Regulation Goals

We found that emotion regulation goals differ dramatically in their frequencies. The most prevalent goal is *to feel better* (in about every 2.5 events). Further, to keep up appearances and to avoid conflict (in about every third or fourth events) occur slightly less often than to feel better. To make others feel better and to influence others occur the least often (in about every tenth to fiftieth event). Participants reported to make others feel better and to influence others infrequently. One potential explanation is that to make others feel better may be more important in older samples with caring responsibilities (e.g. young children or care of elderly relatives). Similarly, to influence others may be more important in a professional or occupational context. Alternatively, participants could have been reluctant to acknowledge that they want to influence others, since it may be interpreted as socially undesirable. This may explain why we observe these emotion regulation goals relatively infrequently.

In comparison to prior daily diary studies (English et al., 2017; Kalokerinos et al., 2017c), we found lower frequencies in emotion regulation goals. Kalokerinos et al. (2017c) found social motives about once in 10 events, but they used a very broad item. English et al. (2017) used more specific items and found prohedonic goals about every second event, *to avoid conflict* about every third, *to keep up appearances* about every two and a half events, and *to make others feel better* in about every fifth event. However, these studies asked participants only once a day about one emotional situation. We attribute these differences found in our study to its higher resolution. It makes sense that we found lower frequencies, as the likelihood of having a negative emotion event over the course of a day is higher than over the course of 3 h.

Further, we found substantial differences in the ICCs of emotion regulation goals with 0.21, 0.11, 0.14, 0.09, and 0.02 for *to feel better*, *to avoid conflict with others*, *to keep up appearances*, *to make others feel better*, and *to influence others*, respectively. Participants reported *to make others feel better* and *to influence others* infrequently.

In comparison to previous studies (English et al., 2017; Kalokerinos et al., 2017b; Eldesouky and English, 2018), we found lower ICCs in emotion regulation goals. Eldesouky and English (2018) found ICCs between 0.57 and 0.87. English et al. (2017) reported ICCs between 0.18 and 0.38. Kalokerinos et al. (2017a) found ICCs between 0.15 and 0.29. The difference to our study may be attributable to the higher resolution – the more surveys a day, the higher the within-variance (i.e., the lower the ICC) (Podsakoff et al., 2019). Moreover, lower ICCs may also reflect that the current study captures a larger variety of different situations. Prior studies asked about the most negative event of the day, whereas we asked for the most negative event of the last 3 h. Thus, the chance of capturing a situation with lower emotional intensity is higher in our study, and participants may be less motivated to develop an emotion regulation goal for less emotionally intense events (Milyavsky et al., 2019). Emotion regulation goals may vary more strongly among different events for this reason.

A recent meta-analysis examined the ICCs of a wide range of psychological constructs and found an average ICC of about 0.52 (Podsakoff et al., 2019). Accordingly, emotion regulation goals varied considerably more among different situations in our sample, compared to other constructs.

#### Situational Factors

We found that situational factors differ in their average degree of expression. In descending order, expected reoccurrence, emotional intensity, and perceived control show the strongest average degree of expression in negative emotion events. Compared to another study (Haines et al., 2016), we observed a slightly lower level of perceived controllability in negative emotion events. We are not aware of any study that examined emotional intensity or expected reoccurrence in the context of emotion regulation in an experience sampling study.

Further, we found substantial differences in the ICCs. In descending order, we found 0.28, 0.22, and 0.17 for perceived control, expected reoccurrence, and emotional intensity, respectively. Haines et al. (2016) reported an ICC of 0.35 for perceived control, which is slightly above the value found in this study, but they conducted a two-level model (events nested in persons) for their estimation. Our value may be lower because we used a three-level model instead.

Comparing the situational factors’ ICCs to the average ICC among psychological constructs (see above; Podsakoff et al., 2019), situational factors varied considerably more among different situations in our sample, compared to other constructs.

#### Conclusion: How Often and How Consistently Do Predictors for Emotion Regulation Choice Occur in Daily Life?

All in all, we conclude that certain emotion regulation goals and situational factors are more prevalent than others in negative emotion events. Further, our results suggest that emotion regulation goals and situational factors should be treated as states, but not as dispositional personality traits, as they vary strongly among different events. Overall, our results are consistent with the emotion regulation flexibility framework: Individuals regulate their emotions in vastly different contexts (Aldao, 2013; Bonanno and Burton, 2013; Aldao et al., 2015).

### What Predicts Emotion Regulation Choice in Daily Life?

#### Emotion Regulation Goals

Emotion regulation goals were associated with different emotion regulation strategies. Prohedonic goals were positively associated with the use of active coping, cognitive reappraisal, and distraction, but not with expressive suppression. Our results are broadly consistent with the notion that individuals use emotion regulation strategies that are functional for their goals (Tamir et al., 2015; Eldesouky and English, 2018; Milyavsky et al., 2019). The inclusion of active coping is particularly notable, as it is an understudied strategy.

We found small effect sizes for active coping and cognitive reappraisal, and a medium effect size for distraction. Our results suggest that participants relied more strongly on strategies, which have less cognitive costs. This is in line with the cognitive energetics theory (Kruglanski et al., 2012), which posits that individuals prefer to use emotion regulation strategies that consume less cognitive energy to achieve their goals (Milyavsky et al., 2019).

However, prohedonic goals were positively associated with rumination. This appears to be inconsistent with the notion of a functional association between emotion regulation goals and emotion regulation strategies, but individuals often use rumination to better understand a critical situation, to gain insight, and to solve problems (Nolen-Hoeksema et al., 2008). We could conclude that participants often falsely believe that it will help them to feel better. Alternatively, some kinds of repetitive thoughts are actually constructive (for a review, see Watkins, 2008), for example, when they are more concrete instead of abstract. Since we did not distinguish between concrete and abstract forms of rumination, our item may have captured both. The association between prohedonic goals with a concrete form of rumination may be interpreted as functional, whereas the association with an abstract form of rumination may be interpreted as dysfunctional. However, other studies used a similarly broad item and found that rumination was associated with longer periods of higher negative emotional intensity (Kalokerinos et al., 2017b; Résibois et al., 2018), and with a more explosive trajectory of negative emotional intensity (Résibois et al., 2018). We are therefore inclined to interpret our results as a dysfunctional link between prohedonic goals and rumination, although more evidence is required for a final conclusion.

*To keep up appearances* and *to avoid conflict* were positively associated with expressive suppression and distraction. Our results suggest a functional association between social goals and emotion regulation strategies, which is largely consistent with other studies (e.g., English et al., 2017; Eldesouky and English, 2018). Participants used strategies that change the immediate expression or experience of an emotion for social goals more often. Since the interaction with another person probably occupies some cognitive resources, participants may have preferred low-cost distraction over high-cost active coping and cognitive reappraisal (Sheppes et al., 2014). For example, when an individual wants to hide an emotion, while talking to a friend, the conversation consumes some of the cognitive resources. Additionally, she could engage in distraction for highly intense emotions, when they may not be able to, or it may be too costly to suppress she emotional expression.

We found a small effect size for distraction and a medium effect size for expressive suppression: Hiding emotional expressions may be more important for these goals than reducing emotional experiences. Our results suggest that participants may have sometimes wanted to maintain (or not alter) a negative emotion, when they pursue social goals, but simply hide them from others.

*To influence others* was not negatively associated with expressive suppression. Generally, participants reported *to influence others* extremely seldom. We therefore hesitate to interpret the results for this goal.

##### Conclusion for emotion regulation goals

All in all, we conclude that our results largely support the notion of the functional association between prohedonic goals, but also social goals, and emotion regulation strategies. Emotion regulation strategies, effective in changing the emotional experience, appear to be particularly important for prohedonic goals, whereas expressive suppression appears to be particularly important for social goals. By contrast, the positive association between prohedonic goals and rumination seems to be dysfunctional. In this case, our results do not support the notion of the functional association between emotion regulation goals and strategies.

#### Situational Factors

Situational factors were associated with different emotion regulation strategies. Perceived control was positively associated with active coping, and unexpectedly positively with cognitive reappraisal, each with small effect sizes. In more controllable situations, participants relied on emotion regulation strategies that change either the actual situation or the appraisal of that situation, which partly contradicts our predictions. One potential explanation is that active coping and cognitive reappraisal occurred together sometimes (*r*_within_ = 0.20; *p* < 0.001). For example, participants may have used cognitive reappraisal first to gain or maintain confidence and then deal with the situation actively, or first actively deal with the situation and then change their appraisal on the situation. Overall, our results suggest that perceived control appears to increase the use of putatively adaptive strategies.

Emotional intensity was positively associated with rumination with a medium effect size, which replicates the results from Dixon-Gordon et al. (2015). This result suggests that high emotional intensity triggers the use of rumination. As outlined above, rumination does not necessarily need to be a dysfunctional strategy to regulate emotions (Watkins, 2008), but other studies found that rumination (measured with a similar item) was positively associated with the accumulation of negative emotional intensity (Kalokerinos et al., 2017b; Résibois et al., 2018). Accordingly, if negative emotional intensity is high, the use of rumination preserves this state, which we interpret as a dysfunctional emotion regulation choice in most cases. Alternatively, the result may indicate a mutual reinforcement of emotional intensity and rumination, since rumination also increases the emotional intensity of an event (Kalokerinos et al., 2017b). All in all, this result suggests a dysfunctional association between emotional intensity and rumination.

Emotional intensity was negatively associated with cognitive reappraisal with a small effect size. Several experiments validated: The higher the emotional intensity, the lower the frequency of cognitive reappraisal (Sheppes et al., 2011, 2014). We complement this research by supporting this finding in everyday life.

Unexpectedly, emotional intensity was negatively associated with active coping with a small effect size. A previous study, in which individuals were asked to recall events from the past, found a positive association between emotional intensity and active coping instead (Dixon-Gordon et al., 2015). Our results contradict their findings. The difference could be explained because people recall memories that are short or long in the past with different accuracy.

Likewise, emotional intensity was not associated with distraction. Several experiments validated: The higher the emotional intensity, the more frequently individuals use distraction (Sheppes et al., 2011, 2014). Our experience sampling study does not support the findings from the laboratory and suggests that participants regulated their emotions differently in everyday life. Since the main effect of emotional intensity on distraction was replicated in a German sample (Scheibe et al., 2015), it also appears unlikely that cultural differences caused the observed difference between the laboratory studies and our experience sample study. Overall, our results suggest that emotional intensity appears to reduce the use of putatively adaptive strategies, but to increase the use of maladaptive strategies.

Expected reoccurrence was negatively associated with active coping and cognitive reappraisal with small effect sizes, and not negatively associated with distraction. This contradicts our predictions and broadly the results found in the laboratory (Sheppes et al., 2014). One potential explanation for our results may be temporal discounting (for a review, see Frederick et al., 2002): Individuals increasingly discount the value of an event the further in the future it takes place. In the laboratory, individuals may discount the reoccurrence of a negative stimulus less than in everyday life because they expect to encounter it again sooner (i.e., during the laboratory session). They thus may be more motivated to engage in strategies with long-term effects in the laboratory, but not in everyday life. Alternatively, participants may have assumed that a similar situation reoccurs in the future, but they were unable to act on it immediately in a reasonable way. For example, a student fails an exam, and the next chance to take the exam is 1 year. The student may struggle to act immediately on this event. Overall, our results suggest that expected reoccurrence appears to reduce the use of putatively adaptive strategies.

##### Conclusion for situational factors

Most of our hypotheses on situational factors were not supported (6 out of 9). We based five of our hypotheses (H11, H12, H14, H16, and H17) on the results of previous laboratory studies (Sheppes et al., 2011, 2014; Milyavsky et al., 2019; Van Bockstaele et al., 2019) and only replicated the relationship between emotional intensity and cognitive reappraisal. We therefore conclude that participants regulated their negative emotions in largely different ways in the laboratory as compared with everyday life, which underscores the importance of experience sampling studies to better understand emotion regulation. All in all, perceived control was positively associated with putatively adaptive strategies, whereas expected reoccurrence was negatively associated with them. Emotional intensity appears to be positively associated with dysfunctional emotion regulation choice.

#### Conclusion: What Predicts Emotion Regulation Choice in Daily Life?

Consistent with the emotion regulation flexibility framework (Bonanno and Burton, 2013; Aldao et al., 2015), we firstly found a considerable within-person variability in emotion regulation strategies (i.e., ICCs from 0.19 to 0.35). Secondly, we found that different contexts (i.e., emotion regulation goals and situational factors) were associated with different emotion regulation strategies, which suggests that individuals change their emotion regulation as a function of context. However, the majority of effects were small, suggesting either that there are omitted factors, which influence emotion regulation choice or some inherent variability, which is independent from the context.

Hence, we found that emotion regulation goals and perceived control appear to be related to mostly functional emotion regulation choices. By contrast, emotional intensity and expected reoccurrence appear to be related to dysfunctional emotion regulation choice, which does not support findings from the laboratory. Possibly, laboratory stimuli may be easier to handle than the stimuli in participants’ everyday life.

### How Do Predictors for Emotion Regulation Choice Interact in Daily Life?

We did not find any significant interaction effect in this study. One interpretation of this finding is that the interaction effects do not exist, and only the main effects influence emotion regulation choice. However, interaction effects may be hard to find: The measurement error accumulates in the interaction effect, which reduces its effect size (for a similar argument for the expectancy-value model, see Nagengast et al., 2011).

### Limitations and Direction for Future Research

The study has some limitations. First and foremost, we examined emotion regulation goals and situational factors in response to negative events only and therefore cannot draw any conclusions about them in response to positive events. Future studies may address this gap to extend our results to positive events.

Second, we only examined prohedonic and social goals. Examining performance goals, eudaimonic goals, and epistemic goals (Tamir, 2016) would allow better mapping of the frequencies of emotion regulation goals and their (potential) functional or dysfunctional associations with emotion regulation strategies in daily life.

Third, we relied on undergraduate students. Accordingly, it is unclear to what extent our results generalize to other populations. For example, some evidence exists that older individuals pursue prohedonic goals more often than younger ones (Riediger et al., 2009, 2014). Our results would probably look partly different if evaluated in an older sample. Other studies may examine the frequencies of emotion regulation goals and situational factors and their association with emotion regulation strategies in older, less educated samples or working samples. They could then test whether our results can be generalized or are specific to our sample.

Fourth, we relied on non-random sampling, and thus may have over-sampled or under-sampled specific characteristics of the student population. Potentially, the students included in our study (i.e., mostly business-related studies) do not represent the entire student population. It is unclear to what extent our results generalize to the student population in Germany.

Fifth, we relied on a deterministic sampling throughout the day; participants potentially anticipate the scheduled survey and may adjust their momentary experience before it (Beal and Weiss, 2003). Further, negative events could be more likely directly after participants answered a scheduled survey (e.g., because of specific circadian rhythm), which would increase the time between the event participants’ report and the report itself. Hence, since we allowed some delay to answer the prompt, our participants potentially did not answer immediately after a negative event, but instead when they had calmed down. All these factors could introduce bias to their responses (Bolger et al., 2003), although we do not assume this effect to be large. All in all, the deterministic sampling approach, which we chose to reduce the burden of participants, diminishes the ecological validity of our results. Future studies may benefit from random sampling over the day (Brans et al., 2013) to capture the emotional life of participants more naturally.

Finally, the examined relationships between emotion regulation goals and emotion regulation strategies, and between situational factors and emotion regulation strategies were correlational in nature: A causal conclusion should not be drawn from the data. It is likely that some of the examined relationships are bidirectional, but experimental designs are needed to disentangle the directionality and show causal relationships.

## Conclusion

Overall, the findings from this study suggest that, firstly, emotion regulation goals and situational factors are very context-dependent; they should be treated as states, instead of traits. Secondly, emotion regulation goals and perceived control appear to be related to mostly functional emotion regulation choices. In contrast, emotional intensity and expected reoccurrence appear to be related to dysfunctional emotion regulation choices in daily life. These associations differ largely from the results found in the laboratory, which underlines the importance of experience sampling studies. Finally, the interactions of emotion regulation goals and situational factors need further investigation in future studies.

## Data Availability Statement

All datasets generated for this study are included in the article/Supplementary Material.

## Ethics Statement

Ethical approval was not provided for this study on human participants because the used questions are not of ethical concern, the data were stored anonymously, and participants had the chance and possibility to quit the study at any time without further consequences. In addition, people had to give their consent to participate in the study, and only then, we used their data for scientific research. The study was not approved by any other ethics committee, as the design of the study is common practice. Following §7.3.1 of the “Ethical guidelines, The Association of German Professional Psychologists” the approval by an ethical committee is optional. The patients/participants provided their written informed consent to participate in this study.

## Author Contributions

RW designed the concept of manuscript, supervised the data gathering process, analyzed, and interpreted the data, and drafted the manuscript. RL and AK contributed substantially in the process by providing feedback at all stages of the research and the manuscript. All authors contributed to, reviewed, and commented on drafts of the manuscript. All authors read and approved the final manuscript.

## Conflict of Interest

The authors declare that the research was conducted in the absence of any commercial or financial relationships that could be construed as a potential conflict of interest.

## Footnotes

1 The group-mean first creates the mean for each person (person-mean) separately and afterward calculates the mean of the person-means.

## Appendix

**TABLE 1:** Within-correlations of the predictors.

	**1**	**2**	**3**	**4**	**5**	**6**	**7**	**8**	**9**
1. To avoid conflict	1.00								
2. To keep up appearances	0.13^∗∗^	1.00							
3. To feel better	–0.18^∗∗∗^	0.04	1.00						
4. To influence others	0.07	0.02	–0.06	1.00					
5. To make someone else feel better	0.17^∗∗∗^	0.02	–0.03	0.04	1.00				
6. To work or learn	0.02	0.00	–0.02	–0.05	−0.08^∗^	1.00			
7. Perceived control	0.07	0.05	0.02	–0.02	0.07	0.01	1.00		
8. Emotional intensity	–0.07	–0.05	0.10^∗^	0.04	–0.03	0.05	–0.12^∗∗^	1.00	
9. Expected reoccurrence	–0.01	0.00	0.00	0.01	–0.02	0.07	0.11^∗∗^	0.08	1.00

*^∗^p < 0.05; ^∗∗^p < 0.01; ^∗∗∗^p < 0.001.*
